# Flagellar Motility Is Critical for *Salmonella enterica* Serovar Typhimurium Biofilm Development

**DOI:** 10.3389/fmicb.2020.01695

**Published:** 2020-09-09

**Authors:** Feiying Wang, Le Deng, Fangfang Huang, Zefeng Wang, Qiujun Lu, Chenran Xu

**Affiliations:** State Key Laboratory of Developmental Biology of Freshwater Fish, College of Life Science, Hunan Normal University, Changsha, China

**Keywords:** flagellum, biofilms, motility, *S.* Typhimurium, confocal laser scanning microscopy

## Abstract

The food-borne pathogen *Salmonella enterica* serovar Typhimurium (*S.* Typhimurium) causes self-limiting gastroenteritis in humans and is not easily eradicated because it often attaches to suitable surfaces to form biofilms that have high resistance to disinfectants and antimicrobials. To develop an alternative strategy for the treatment of biofilms, it is necessary to further explore the effects of flagellar motility on the development process of *Salmonella* biofilms. Here, we constructed flagella mutants (Δ*flgE* and Δ*fliC*) to systematically study this process. By comparing them with wild-type strains, we found that these mutants lacking flagellar motility form fewer biofilms in the early stage, and the formed mature biofilms contain more cells and extracellular polymeric substances (EPS). In addition, fewer mutant cells adhered to glass plates compared with wild-type cells even after 6 h of incubation, suggesting that flagellar motility plays a significant role in preliminary cell-surface interactions. More importantly, the motility of wild-type strain was greatly decreased when they were treated with carbonyl cyanide m-chlorophenylhydrazone, which inhibited flagellar motility and reduced biofilm formation, as in the case of the Δ*flgE* mutant. Overall, these findings suggest that flagellar motility plays an important role in *Salmonella* biofilm initiation and maturation, which can help us to counteract the mechanisms involved in biofilm formation and to develop more rational control strategies.

## Introduction

Contamination of fresh produce with food-borne pathogens is a potential threat to public health. *Salmonella enterica* serovar Typhimurium (*S.* Typhimurium) is a food-borne pathogen that causes high morbidity worldwide, including life-threatening infections in fetuses, newborns, and immunocompromised individuals (Achinas et al., 2019). Its natural habitat is the gut of animals and humans, and infection occurs through fecal contamination of soil, water, plants and medical equipment, which spreads the bacteria to other hosts (Semenov et al., 2010; Jacobsen and Bech, 2012; MacKenzie et al., 2017). Once attached to one of these surfaces, the bacteria begin to form biofilms. Bacteria inside biofilms are more resistant to external antibiotics, disinfectants, and environmental stress, so they can be extremely difficult to eradicate, in contrast to planktonic bacteria (Pande et al., 2016). The biofilms cause food safety issues and can lead to enormous economic losses.

Biofilms are the preferred lifestyle for most microorganisms in nature (Stoodley et al., 2002). Biofilms begin to develop when bacteria adhere reversibly to a surface. The adhered cells then form microcolonies, which synthesize extracellular matrices to support mature three-dimensional biofilms. Bacteria within the biofilms can undergo controlled dissociation, resulting in biofilm dispersal (Watnick and Kolter, 2000; Koo et al., 2017). Biofilm formation is influenced by the structural composition (curli and other fimbriae, flagella, BapA et al.), the bacterial genome, environmental signals, and stress factors (Watnick and Kolter, 2000; Simm et al., 2014; MacKenzie et al., 2017). A variety of biofilms inhibitors have been developed that target these factors, including some that target the flagella. Previous research has shown that furanones reduce biofilm formation through interfering with *Salmonella* flagellar synthesis (Janssens et al., 2008). Moreover, zinc is required for flagellar expression, and the absence of flagella impairs the ability of *S.* Typhimurium to produce biofilms (Ammendola et al., 2016). However, in order to make more effective strategies for the treatment of biofilms targeting flagella, it is necessary to further clarify the effect of flagellar motility on the development process and composition of *Salmonella* biofilms.

Flagella, as main motility organs of bacteria, play important roles in the formation biofilm of several gram-negative bacteria (O’Toole et al., 2000; Haiko and Westerlund-Wikström, 2013). Some studies have proposed that flagella might act in biofilm formation both as providers of motility and as surface adhesins, however, in *Escherichia coli* and *Listeria monocytogenes* it is motility itself that is critical (O’Toole and Kolter, 1998; Pratt and Kolter, 1998; Lemon et al., 2007). In *S.* Typhimurium, previous studies performed with *motA* and *fliA* mutants have suggested that motility is necessary for biofilm development on the glass (Huber et al., 2002; Prouty and Gunn, 2003). Moreover, it has been described that *Salmonella* mutants with defective flagella (*flhC* or *flgE*) are unable to develop complete biofilms in the presence of bile (Crawford et al., 2010; Tsai et al., 2019). Previously, work regarding QseBC two-component system (TCS) function in *S.* Typhimurium revealed that QseBC functions as a global regulator of flagella, biofilm formation, and virulence (Merighi et al., 2009; Bearson et al., 2010; Ji et al., 2017). Recent studies have shown that the cyclic di-guanylate monophosphate (c-di-GMP) receptor YcgR and the phosphodiesterase YhjH can distinctively inhibit flagellar motility in *S.* Typhimurium (Le Guyon et al., 2015; Han and Yoon, 2019). Taken together, the role of flagellar motility in the development of *Salmonella* biofilm is very important. By observing the number and distribution of polysaccharides and cells, we can more intuitively judge the effect of flagellar motility on biofilm initiation and maturation. Therefore, this study aims to deepen the understanding of the effect of flagellar motility on biofilm development and to provide theoretical support for the development of biofilms inhibitors that interfere with flagella motility.

In many motile bacteria, the synthesis and assembly of flagella involve at least 50 genes that are divided into early, middle, and late genes, which are hierarchically and temporally synchronized (Aizawa, 1996). *flgE* is a middle gene that encodes the major component of the hook-basal body structure, which is necessary for flagellar filament elongation (Bonifield et al., 2000). *fliC* is a late gene that encodes the major component of the flagellin structure, which is necessary for the formation of the helical filament (Chilcott and Hughes, 2000). Mutations in these two genes may impair flagellar motility, therefore, our primary approach involved deleting *flgE* and *fliC* and observing the effect on flagellar motility. Our results confirmed that a lack of flagellar motility affects bacterial contact with abiotic surfaces, and reduces cell colonization and early biofilm formation. Moreover, the mature biofilms formed by these flagella mutants had denser matrices and contained more aggregated cells.

## Experimental

### Bacterial Strains, Plasmids, and Growth Media, and Chemicals

*S.* Typhimurium CMCC 50115 is a *S.* Typhimurium LT2 derivative. All strains and plasmids used in this work are described in Supplementary Table S1. All strains were grown on Luria-Bertani (LB) broth agar plates or in LB broth liquid medium (Tryptone 10 g/L; Yeast Extract 5 g/L; NaCl 10 g/L) at 37°C. Chloramphenicol (Cm, 25 μg/mL), ampicillin (100 μg/mL), L − (+)− Arabinose (5 mmol/L) were added when required. SYTO^®^ 9 (Invitrogen, S 34854); Alexa Fluor^®^ 647 conjugate of Con A (Invitrogen, C 21421); Carbonyl cyanide m-chlorophenylhydrazone (CCCP, Sigma) acted as an inhibitor of proton-driven force of the flagella (Kosaka et al., 2019).

### Rdar Morphotype and Pellicle Formation

After one colony of the bacteria from LB plates was transferred into a tube containing 3 mL of LB medium and incubated for 18 h at 37°C, the amount of inoculation was adjusted to optical density OD_600_ = 1.0, and confirmed by plate counts of 10 fold dilutions of the bacteria. These bacterial cultures contained approximately 10^8^CFU/mL. The *Salmonella* morphotype was judged visually on Congo red agar plates, 2 μL of bacterial suspension (OD_600_ of 4.0) was plated onto LB agar plates without NaCl and complemented with Congo red (40 mg/L, Aladdin) and Coomassie brilliant blue G-250 (20 mg/L, Sangon). The inoculated plates were incubated at 28°C for 48 h and colonies were visualized macroscopically.

The method measures biofilm production on the air-liquid interface in a modified method as previously reported (Ramachandran et al., 2016). Briefly, overnight *Salmonella* strains were transferred into 3 mL of LB medium without NaCl (1:100 dilutions) for 5 days at 28°C without shaking. To observe the biofilms that form at the liquid-air interface, the culture was gently poured out and further stained with 1% crystal violet.

### Biofilm Formation and Quantification Assay

Biofilm formation was observed as described previously with some modifications (Baugh et al., 2012). Experiments in 96-well polystyrene microtiter plates (Sangon) were performed. Overnight cultures were diluted to 10^6^ CFU/mL in LB medium without NaCl. A total volume of 0.2 mL was added per well, followed by 24 h incubation at 28°C in static conditions. The total amount of biofilm biomass was quantified by crystal violet. After careful removal of the medium from wells, the biofilms were rinsed three with deionized water. 1% crystal violet was used to stain biofilms for 15 min, and then the excess of stain was removed by gently washing with deionized water. Residual crystal violet was solubilized with 200 μL of 33% glacial acetic acid per well and the OD_595_ was measured using an ELISA reader (Thermo Scientific Labsystems 354). LB medium without NaCl was used as a negative control in all biofilm assays. All experiments were repeated three times at different time points.

### Construction of Mutants and the Complemented Strains

The flagella-deficient strains were constructed by allelic replacement via homologous recombination (Datsenko and Wanner, 2000). Homologous regions and a chloramphenicol cassette with two FRT sites were PCR-amplified from pKD3, and the purified PCR product was digested with *Dpn*I (Invitrogen) and electroporated into bacteria carrying pKD46. Antibiotic selection and the λRed recombineering system led to homologous recombination between the fragments and the host strain genome, and the recombinants were selected for on agar plates containing chloramphenicol. The pCP20 plasmid was introduced to the recombinant strains to remove the DNA fragment containing the chloramphenicol resistance gene, resulting in a single FRT site within the targeted genomic segment. The markerless mutant strains were verified by genomic DNA PCR using primers that annealed to sequences flanking the target gene and further confirmed by sequencing analysis (Supplementary Table S2 and Supplementary Figures S1, S2).

To generate the Δ*flgE* complemented strain, the full-length *flgE* gene was PCR-amplified from *S.* Typhimurium wild-type strain CMCC50115 genomic DNA using the primers *flgE*–F3 and *flgE*–F4 (Supplementary Table S2). The PCR product was ligated into pBad/gIIIA, and the resulting plasmid was transformed into the Δ*flgE* strain by electroporation. The Δ*fliC* complemented strain was constructed by the same method.

### Motility Assays

Swimming motility was performed as described previously (Duan et al., 2012). Briefly, 30 μL cultured overnight of bacterial was re-inoculated into 3 mL of LB and incubated at 37°C with a shaker at 200 rpm until a density value of approximately 1.0 at OD_600_. The culture samples were inoculated onto 0.4% tryptone agar plates as 1 μL aliquots (Tryptone 1%, NaCl 0.5%, Agar 0.4%). The bacteria grew for 12 h at 37°C and the swimming diameter was measured. Photographs were taken by using the SONY Alpha 7 camera.

### Transmission Electron Microscopy (TEM) Analysis

Flagellar morphology was visualized by TEM with negative staining. A single colony was picked and inoculated into fresh LB medium, and the OD_600_ value of the bacteria was adjusted to about 0.5. The cells were washed once with deionized water and resuspended in sterile water. A sample of 5 μL of the bacterial solution was added dropwise to a copper mesh having a carbon film and dried at room temperature. Using 1% phosphotungstic acid (pH 7.4) for negative staining 2 min, excess solution was blotted with filter paper and observed via electron microscopy (TecnaiG2 F20, FEI, America).

### Western Blot Analysis

Flagellar expression using whole-cell lysates was detected by western blot analysis (Shao et al., 2018). Overnight cultures of the tested bacteria were transformed into the fresh LB medium and cultivated an optimal density value of 1.0 at OD_600_. 2 mL cultures were centrifuged at 9000 × *g* for 10 min at 4°C to pellet the bacteria. The bacterial cells from whole-cell lysates were loaded and separated by 12% SDS-PAGE, followed by transfer onto a PVDF membrane (GE Healthcare, Life Science). The blot was incubated with mouse polyclonal antiserum to H1 flagella (1: 2000 dilutions, TBC, China), followed by HRP-conjugated Goat anti-mouse IgG (1: 200 dilutions, Sangon, China) orderly. The signal was detected by Luminata Forte Western HRP Substrate (Millipore). Photographs were taken by using the Tanon 5500 imaging system.

### Confocal Laser Scanning Microscopy (CLSM) Imaging of Biofilms

After being cultured overnight in LB medium in a shaking 37°C incubator, the cultures were taken to dilute to 10^7^ CFU/mL in 50 mL LB medium without NaCl in a 100 mL Conical flask. A sterile glass slide was gently added into the diluted culture and was taken to statically incubate at 28°C (Supplementary Figure S4). After the culture time was terminated, the glass slide with biofilms was carefully taken out with tweezers and placed in a clean culture dish, and the residual culture medium on them was skimmed with deionized water. The glass slide was stained with 100 μL of SYTO 9 (1 μg/mL, green fluorescence, labeled bacterial cells) and Alexa Fluor 647 (50 μg/mL, red fluorescence, labeled EPS) solution for 30 min, in the dark at room temperature. Confocal microscopy images were obtained on a Leica DMi8 microscope by using a 40 × objective. The settings of the confocal microscope were as follows: the excitation/emission of Alexa Fluor 647 and SYTO 9 were 650/668 and 480/500 nm, respectively. Stack images were obtained by scanning the biofilms along the *Z*-axis at 0.5 μm intervals. The confocal images were analyzed using COMSTAT software for simultaneous visualization and quantification of EPS and bacterial cells within intact biofilms (Heydorn et al., 2000). For each image stack, cell biomass (μm^3^/μm^2^) is defined as the percentage of area occupied by cells labeled by SYTO 9 (green fluorescence); EPS biomass (μm^3^/μm^2^) is defined as the percentage of area occupied by EPS labeled by concanavalin A-Alexa Fluor 647 (red fluorescence); Total biomass (μm^3^/μm^2^) is cell biomass plus EPS biomass.

### Statistical Analysis

The statistical significance of numerical data was compared using variance analysis technology. Significant levels of *p* values were reported by the following symbols: ^∗^*p* < 0.05, ^∗∗^*p* < 0.005, and ns, with no difference.

## Results

### *S.* Typhimurium CMCC 50115 Biofilm Production

**FIGURE 1:**
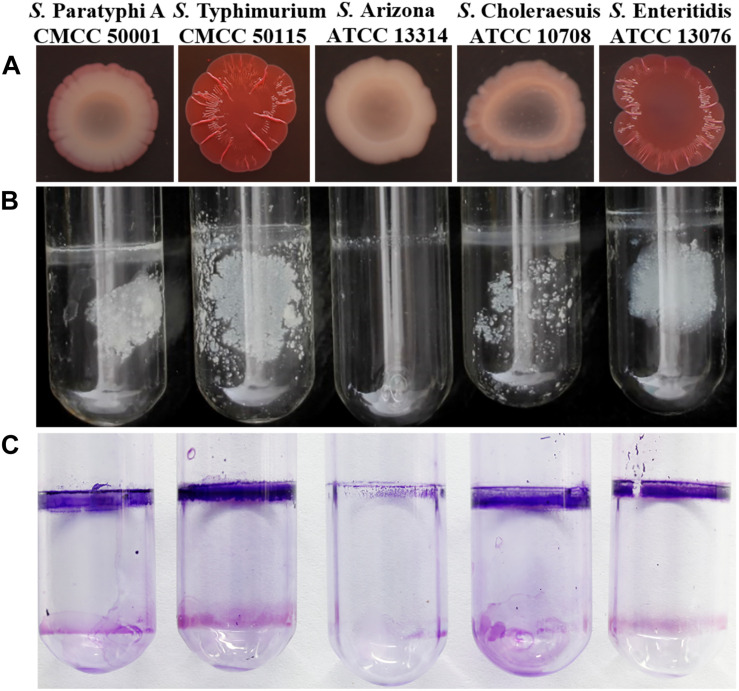
Phenotype of *Salmonella* assay. **(A)** Congo red morphotype assay five *Salmonella* strains; **(B)** Pellicle formation at the liquid-air interface of five *Salmonella* strains; **(C)** Crystal violet stained and observed the formed pellicle of five *Salmonella* strains.

The best-studied *Salmonella* biofilm phenotype is the rdar morphotype (Römling et al., 1998). After culturing on media containing Congo red at 28°C for 2 days, both *S.* Typhimurium CMCC50115 and *S.* Enteritidis ATCC 13076 formed strong rdar morphotypes (Figure 1). In liquid culture, biofilms form a matrix comprising curli and cellulose called pellicles, which appear as films of cell growth at the air-liquid interface (Solano et al., 2002). *S.* Typhimurium and *S.* Enteritidis, but not the other strains, formed obvious pellicles at the liquid-air interface (Figure 1). Since crystal violet staining showed that *S.* Typhimurium CMCC50115 formed more biofilms compared with the other strains (Table 1), we selected it for use as our wild-type control.

**TABLE 1:** Collated biofilm assay results of *Salmonella* tested in this study.

***Salmonella* Serotypes**	**Crystal Violet Assay (OD_595_)**			**Morphotype on Congo red^b^**	**Pellicle formation^c^**
	**LB**	**TSB**	**LB without salt**		
*Salmonella* Typhimurium *CMCC 50115*	0.428 ± 0.131*^*a*^*	0.945 ± 0.232	2.527 ± 0.612	rdar	+++
*Salmonella* Paratyphi A *CMCC 50001*	0.125 ± 0.056	0.545 ± 0.112	1.875 ± 0.423	bdar	+
*Salmonella* Enteritidis *ATCC 13076*	0.319 ± 0.081	0.613 ± 0.153	2.345 ± 0.103	rdar	+++
*Salmonella* Choleraesuis *ATCC 10708*	0.267 ± 0.061	0.322 ± 0.152	1.658 ± 0.064	bdar	++
*Salmonella* Arizona *ATCC 13314*	0.113 ± 0.03	0.214 ± 0.121	0.903 ± 0.032	saw	+

*^*a*^Average OD (595 nm) ± standard error from two separate experiments. ^*b*^rdar: red, dry, and rough morphotype indicating curli and cellulose production; bdar: brown, dry, and rough morphotype indicating curli production but lack of cellulose synthesis; saw: smooth and white morphology. ^*c*^pellicle formation: +++, strong pellicle; ++, moderate pellicle; +, weak pellicle.*

### Construction and Characterization of Flagella Mutants

**FIGURE 2:**
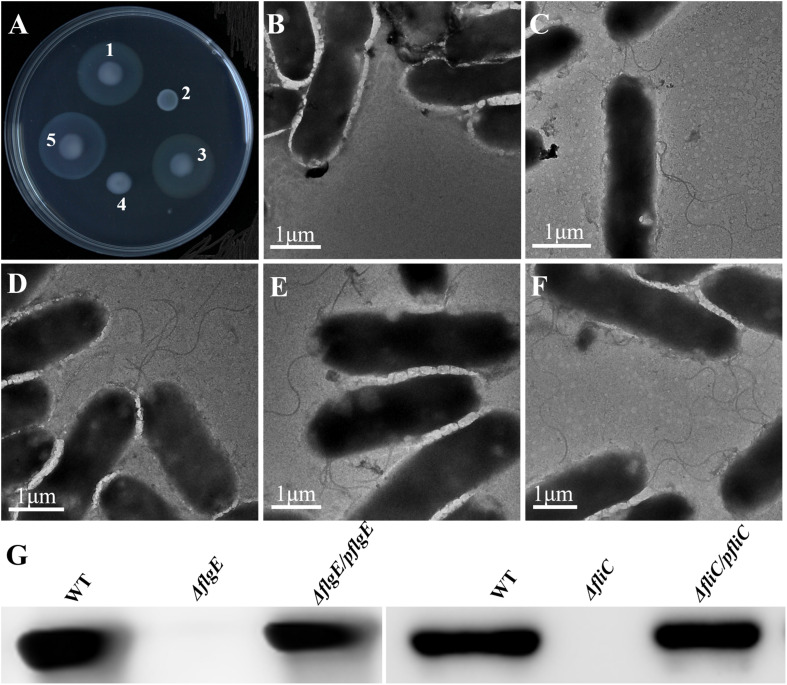
Phenotypes of wild-type *S.* Typhimurium and isogenic mutants. **(A)** Motility of (1) *S.* Typhimurium; (2) Δ*flgE* (*S.* Typhimurium *flgE*^–^); (3) Δ*flgE*/p*flgE* (Δ*flgE* complemented with p*flgE*); (4) Δ*fliC* (*S. Typhimurium fliC*^–^); (5) Δ*fliC/pfliC* (Δ*fliC* complemented with p*fliC*) incubated for 12 h at 37°C. **(B–F)** Expression of flagella by the isogenic mutants was assessed by TEM, **(B)**: Δ*flgE*, **(C)**: Δ*fliC*, **(D)**: WT, **(E)**: Δ*flgE/pflgE*, **(F)**: Δ*fliC/pfliC;* **(G)**: Flagellin deficiency was confirmed by immunoblot analysis with mouse polyclonal antiserum to H1 flagella. WT, wild-type.

The wild-type strain was used to construct the Δ*flgE* and Δ*fliC* isogenic mutants via homologous recombination. The resulting Δ*flgE* and Δ*fliC* mutants exhibited impaired motility on 0.4% tryptone agar plates (Figure 2A). In the case of the Δ*flgE* strain, this may have been because of the loss of flagella. Confirming this, the flagellin protein was not detected by western blot analysis of whole-cell lysates (Figure 2G), and flagella were not observed by TEM in this mutant strain (Figures 2B,D). In contrast, flagellar protein expression (Figure 2G) and flagella production (Figure 2E) was restored in the complemented strain (Δ*flgE*/p*flgE*), which contained a recombinant plasmid expressing FlgE. In the Δ*fliC* strain, the impaired motility phenotype may be related to the absence of the FliC subunit, which plays a significant role in flagellar motility. As *S*. Typhimurium expresses two filament proteins, FliC (H1 flagellin) and FljB (H2 flagellin), the Δ*fliC* mutant could be flagellated under some circumstances. Corroborating this, flagellin protein was not detected by western blot analysis of whole-cell lysates (Figure 2G), but the Δ*fliC* strain had flagella (presumably composed of FljB), as observed by TEM (Figure 2C). In contrast, flagellar protein expression (Figure 2G) and flagella production (Figure 2F) was restored in the complemented strain (Δ*fliC*/p*fliC*), which contained a recombinant plasmid expressing FliC. In addition, it was verified that the *fljB* sequence of the Δ*fliC* strain did not mutate (Supplementary Figure S7). Therefore, the Δ*fliC* strain lacking motility through FljB probably depends on some characteristics of the used strain. Taken together these data show that *flgE* and *fliC* are indispensable for flagellar motility.

### Flagella Mutants Are Defective in the Early Stage of Biofilm Formation

**FIGURE 3:**
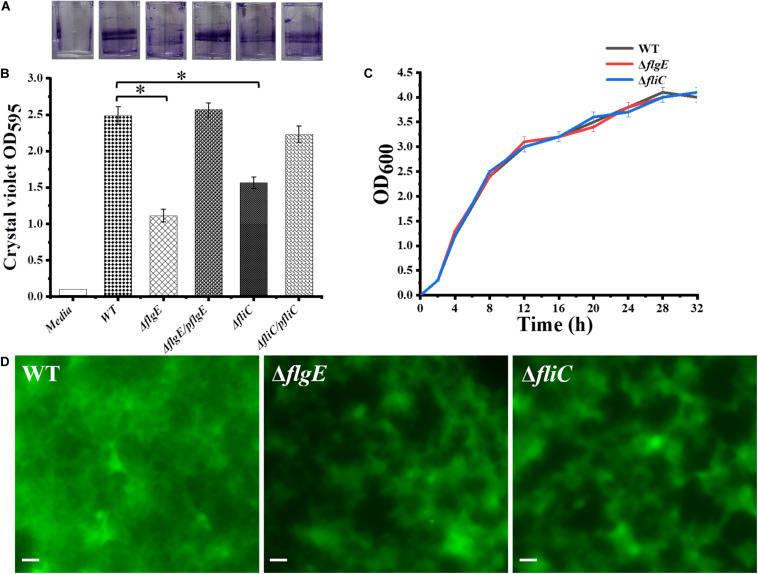
Deletion of the gene encoding flagella was defective in biofilm formation. **(A)** Crystal violet stained *S.* Typhimurium biofilms formed on wells of a 96-well polystyrene plate. The first well has medium alone (Media), followed by the wild-type (WT), *flgE* mutants (Δ*flgE*), Δ*flgE* complemented with p*flgE* (Δ*flgE/pflgE*), *fliC* mutants (Δ*fliC*), Δ*fliC* complemented with p*fliC* (Δ*fliC*/p*fliC*). **(B)** Graph of surface-adhered biofilms 24 h biomass of controls (Media and WT), mutants (Δ*flgE*, Δ*fliC*) and complemented strains (Δ*flgE/pflgE*, Δ*fliC*/p*fliC*) were quantified by staining with cystal violet. The data are averages of four independent experiments and were analyzed using the Student’s two-tailed *t*-test. Error bars represent the standard deviation. **(C)** Growth curves for WT, Δ*fliC*, Δ*flgE*. Bacteria were grown in LB broth at 37°C for 28 h with agitation, and the OD_600_ was determined at hourly intervals. The data are the means of three independent experiments and error bars represent the standard deviation. **(D)** Top-down view of 24 h biofilms stained with SYTO 9 and propidium iodide. Scale bar: 20 μm. ^∗^*p* < 0.05.

The initial attachment of bacteria to a surface is a key step in their ability to form a biofilm. Compared with the wild-type strain, the two flagellar mutants were defective in the early stage of biofilm formation (Figure 3A). We measured biofilm formation by crystal violet staining and found that the two mutants formed fewer biofilms than the wild-type strain, with an average absorbance at 595 nm (OD_595_) of 0.93 ± 0.13 and 1.61 ± 0.21, respectively, compared with an OD_595_ of 2.47 ± 0.16 for the wild-type strain (Figures 3A,B). There was no difference in viability between the flagella mutants and the wild-type strain (Figure 3C). Importantly, biofilm formation was restored to wild-type levels when the mutants complemented (Figures 3A,B). Overall, these data demonstrate that flagella-mediated motility plays an important role in the early stage of biofilm formation.

**FIGURE 4:**
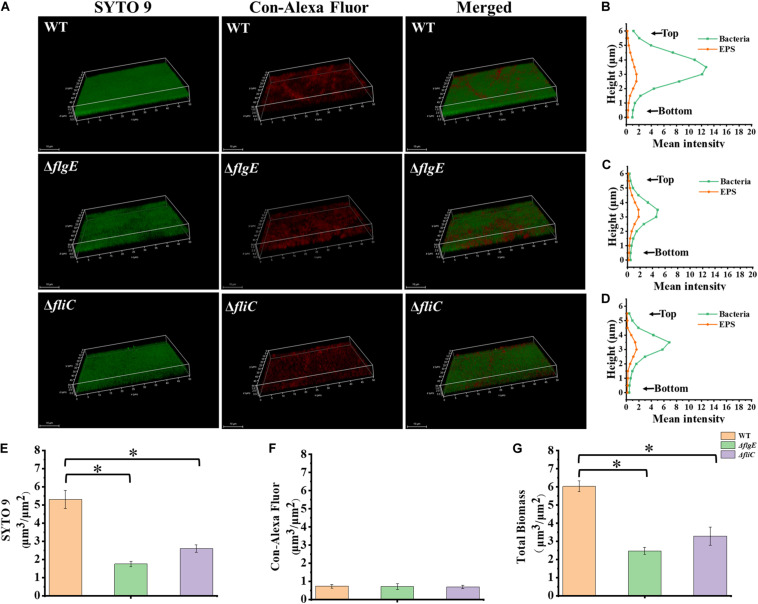
Δ*flgE* and Δ*fliC* biofilms were comprised of less bacterial cells than *S.* Typhimurium wild-type biofilms. **(A)** Δ*flgE*,Δ*fliC*, and wild-type were grown for 24 h on the glass, stained with SYTO 9 (green) and Con-Alexa Fluor (red), and 3D images were acquired by CLSM, Scale bar: 10 μm. **(B–D)**, the bacteria and EPS of biofilm thickness in strains WT, Δ*flgE*, and Δ*fliC* are, respectively, indicated. **(E)** SYTO 9, **(F)** Con-Alexa Fluor, and **(G)** Total biomass were quantified using Comstat 2. Data are averages of three replicates (*n* = 3). Error bars represent the standard deviation. The data were analyzed using the Student’s two-tailed *t*-test. *p < 0.05.

Biofilm architecture can be assessed by staining with SYTO 9 and propidium iodide (Vermilyea et al., 2019). Fluorescence microscopy confirmed that biofilms formed by the Δ*flgE* and Δ*fliC* mutants comprised less biomass than those formed by the wild-type strain (Figures 3D, 4G). Biofilm composition was also evaluated by staining with SYTO 9 (which detects all bacteria) and concanavalin A-Alexa Fluor 647 (which detects EPS). There were significantly fewer bacteria in biofilms formed by the flagella mutants compared with those formed by the wild-type strain (Figure 4E). However, there was no significant difference in EPS content between the biofilms formed by the mutant and wild-type strains (Figure 4F). In addition, there was no significant difference in biofilm thickness between the flagellar mutants and the wild-type strain (Figures 4A–D). Therefore, the reduced biofilm formation exhibited by the flagellar mutants could be due to reduced adhesion of planktonic cells at the initial stage compared with the wild-type strain.

### Flagella Mutants Produce More Thick and Dense Biofilms

**FIGURE 5:**
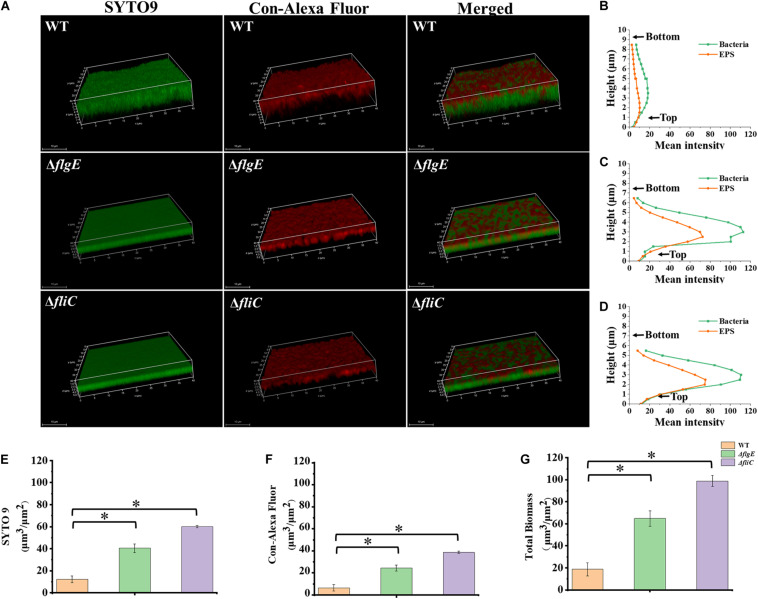
Δ*flgE* and Δ*fliC* biofilms were comprised of more bacterial cells and EPS than *S.* Typhimurium wild-type biofilms. **(A)** Δ*flgE*, Δ*fliC*, and wild-type were grown for 48 h on the glass, stained with SYTO 9 and Con-Alexa Fluor, and 3D images were acquired by CLSM, Scale bar: 10 μm. **(B–D)**, the bacteria and EPS of biofilm thickness in strains WT, Δ*flgE*, and Δ*fliC* are, respectively, indicated. **(E)** SYTO 9, **(F)** Con-Alexa Fluor, and **(G)** Total biomass were quantified using Comstat 2. Data are averages of three replicates (*n* = 3). Error bars represent the standard deviation. The data were analyzed using the Student’s two-tailed *t*-test. *p < 0.05.

To explore the role of flagella on biofilm maturation, CLSM was used to directly observe biofilms that had been allowed to form for 48 h. The wild-type biofilms were twice as thick as the Δ*flgE* biofilms and had a looser inner structure than the mutant biofilms (Figures 5A–D). Additionally, the Δ*flgE* biofilms contained more cells and EPS than the wild-type biofilms (Figures 5E–G). The Δ*fliC* and Δ*flgE* biofilms exhibited similar phenotypes, and flagellar motility was impaired in both strains, suggesting that flagellar motility plays an important role in forming the structure of biofilms. Taken together, these findings indicate that the thick phenotype of biofilms formed by the flagella mutants is due to the accumulation of cells and EPS.

### Flagella Mutants Exhibit Poor Adhesion, Which Inhibits the Initiation of Biofilm Formation

**FIGURE 6:**
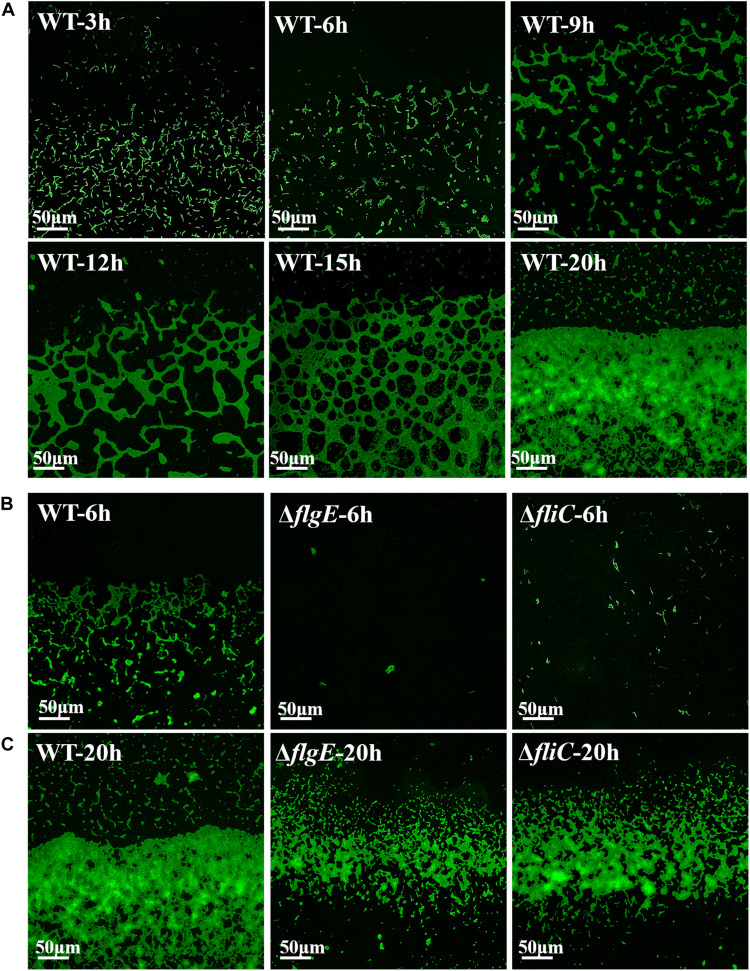
Direct observation of biofilm formation on a sterile glass slide using a confocal fluorescence microscope. **(A)** Observed the adhesion of wild-type biofilms on the sterile glass slide over 20 h. **(B)** Shown are fluorescence micrographs of the wild-type strain, Δ*flgE*, and Δ*fliC* after incubation for 6 h at 28°C. **(C)** The biofilms of the wild-type, Δ*flgE*, and Δ*fliC* at 20 h were displayed. The micrographs were taken at 400 × magnification and the representative fields are shown and the experiments were repeated three times.

Next, we investigated the role of flagellar motility in the early steps in biofilm formation. To assess the initiation of biofilm formation by the flagella mutants and the wild-type strain, we directly visualized biofilm formation on sterile glass slides using a confocal fluorescence microscope. Figure 6A shows a time-course of the development of wild-type biofilms on a sterile glass slide over 20 h at 28°C. Six hours after inoculation, the bacteria had aggregated into small clusters that gradually cross-linked together over the next 6–9 h. By 20 h, the clusters had developed into microcolonies comprising multiple layers of cells.

In contrast, 6 h after inoculation, the Δ*flgE* mutant had only formed a few scattered cell clusters (Figure 6B). By 20 h, the Δ*flgE* strain exhibited fewer colonies and a smaller total attachment area compared with the wild-type strain (Figure 6C). Further statistics on them continually supported the results (Supplementary Figures S5, S6). The Δ*fliC* mutants exhibited similar phenotypes. These observations are consistent with our earlier results showing the importance of flagella-mediated motility in biofilm formation.

### Effect of the Protonophore CCCP on Biofilm Formation

**FIGURE 7:**
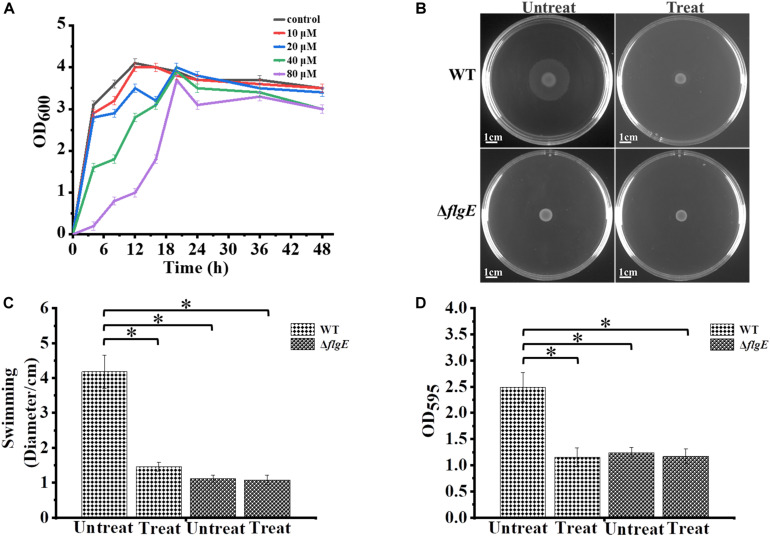
Biofilm formation by *S.* Typhimurium after treatment with CCCP. **(A)** Analysis of the growth curve of wild-type strains treated with different concentrations of CCCP. **(B)** Swimming plates of different groups captured after incubation at 37°C for 12 h. Treat: add 1 μL of 100 mM CCCP to a final concentration of 10 μM CCCP per plate, Untreat: add 1 μL DMSO according to the above treatment. **(C)** The quantitative analysis diameter of swimming. **(D)** The bacteria were cultured with or without 10 μM protonophore CCCP, a quantitative analysis of the formation of biofilms cultured for 24 h by crystal violet staining. Data in **(B–D)** are representative of three independent experiments. Error bars represent the standard deviation. ^∗^*p* < 0.05.

The motility of most bacterial flagella is driven by a proton gradient across the cytoplasmic membrane, so the protonophore CCCP was applied to explore the role of flagellar motility on biofilm formation (Kosaka et al., 2019). First, we treated the wild-type strain with CCCP to determine the optimal concentration for inhibiting cell spreading on semisolid agar. In the presence of 10 μM of the protonophore CCCP on 0.4% tryptone agar plates, the growth of the wild-type strain was not affected (Figure 7A), but its motility was dramatically reduced (Figures 7B,C). Crystal violet staining revealed that the phenotype of the CCCP-treated wild-type strain was consistent with the phenotype of the Δ*flgE* strain (Figure 7D). These results clearly show that flagellar motility plays a key role in *S.* Typhimurium biofilm formation. Treatment with CCCP inhibited the motility of *S.* Typhimurium flagella and thereby reduced the formation of biofilms. Similar results were obtained when *Erwinia carotovora* subsp. carotovora was treated with CCCP, indicating that the role of flagellar motility in biofilm formation is relatively conserved (Hossain and Tsuyumu, 2006).

## Discussion

*Salmonella* is an important food-borne pathogen found all over the world. It has been implicated in the etiology of life-threatening diarrheal diseases, partially due to its ability to colonize and form biofilms on various equipment used in the food production industry (Waldner et al., 2012). Our findings show that the impaired flagellar motility mutant exhibits poor adhesion and smaller colonies in initial biofilms. However, the mutant has a denser and flatter inner structure in mature biofilms. In *E. coli*, flagellar motility is essential for overcoming electrostatic repulsion and tethering cells together during biofilm formation (Pratt and Kolter, 1998; Walker et al., 2004; Serra et al., 2013). Furthermore, high motility leads to vertical structures and low motility leads to flatter microcolonies in mature biofilm architecture (Wood et al., 2006). Our results also confirm that the flagellar motility and biofilm machinery of *S.* Typhimurium and *E. coli* are very similar.

Previous studies have shown that the behavior of a bacterial community at an air-liquid, surface-liquid, or cell-liquid interface changes in the absence of flagella (Römling and Rohde, 1999). Additionally, the flagellar filament (containing the FliC and FljB subunits), and specifically the FliC subunit, of serovar Typhimurium is necessary and specific for cholesterol binding during biofilm initiation (Crawford et al., 2010). Our results show that flagellar motility is impaired in the Δ*fliC* strain, which exhibits a similar phenotype to the Δ*flgE* strain (which is aflagellate). Therefore, it was speculated that the FliC-type filament may play a key role in biofilm formation on abiotic surfaces. The Δ*fliC also* showed a somewhat intermediate biofilm phenotype between the WT and Δ*flgE*, which may be the compensation effect of the *fljB* subunit. Detailed observation by confocal fluorescence microscopy revealed that the flagella mutants (Δ*flgE* and Δ*fliC*) formed mature biofilms with increased content of aggregated cells and EPS compared with biofilms formed by the wild-type strain. It appears that motility and extracellular matrix production are mutually exclusive processes, as many motile bacteria only begin to produce matrix once they have made contact with a surface (Kolter and Greenberg, 2006). We speculate that this may also be true for *Salmonella*, although the specific mechanism regulating this switch requires further study.

Biofilm formation is a complex process that is affected by environmental conditions, gene expression profiles, and physiological shifts within the cell (Mireles et al., 2001). Modulating some of these factors could help prevent biofilm formation. Our results confirmed that flagella-mediated motility is very important in early biofilm formation by *S.* Typhimurium. Our observations regarding the early stages of biofilm development help clarify the mechanisms involved in biofilm resistance and hold promise for developing more effective control strategies for use in the food industry. For example, aptamers or zinc targeting flagella motility (Nielubowicz et al., 2010; Ning et al., 2015; Ammendola et al., 2016), anti-adhesive materials, and the combination of some anti-adhesion materials with antibiotics could be used to control biofilm formation. The results regarding the composition of mature biofilms help us make methods to clear them. Finally, the methods might be directed toward the degradation of the EPS and kill the bacteria in biofilms, which can achieve better therapeutic effects.

## Conclusion

In summary, the formation of *Salmonella* biofilms is a threat to food safety and public health. Understanding the mechanisms underlying biofilm formation will help identify more effective means of treating and preventing the problems it causes. In this study, we constructed Δ*flgE* and Δ*fliC* mutants to further explore the development process of *Salmonella* biofilms. It has been well proved by a series of experiments that impaired flagellar motility mutants exhibit poor adhesion and small colonies in initial biofilms, conversely, they have more dense and complex structures in their mature biofilms. In the near future, flagellar motility could be inhibited to help eliminate biofilms, but it is necessary to select the appropriate treatment time to achieve the desired therapeutic effect.

## Data Availability Statement

The raw data supporting the conclusions of this article will be made available by the authors, without undue reservation, to any qualified researcher.

## Author Contributions

FW designed, supervised the experiments, analyzed the results, revised the first draft, and prepared the last draft of the manuscript. LD performed part of the experiments, contributed significantly to analysis and manuscript preparation, and revised the last draft of the manuscript. FH performed part of the experiments, analyzed the data, and participated in the first draft of the manuscript. ZW and QL analyzed the data and helped perform the analysis with constructive discussions. CX collaborated in the design of the experiments and revised different versions of the manuscript. All authors contributed to the article and approved the submitted version.

## Conflict of Interest

The authors declare that the research was conducted in the absence of any commercial or financial relationships that could be construed as a potential conflict of interest.
