# Supplementary material for: Flagellar Motility Is Critical for Salmonella enterica Serovar Typhimurium Biofilm Development
Source: Front Microbiol. 2020 Sep 9;11:1695. doi: 10.3389/fmicb.2020.01695 (PMC7509047; doi:10.3389/fmicb.2020.01695)
Supplement: Supplementary file 1 [file Table_1.DOCX]

**Table S 1 Strains and plasmids used in this study**

| **Strain(s) or Plasmid** | **Description** | **Source or Reference** |
| --- | --- | --- |
| ***E.* *coli* strains** |  |  |
| **DH5α** | a chemically competent cell strain for molecular cloning | Lab stock |
| ***S.* Typhimurium strains** |  |  |
| Wild–type | a *Salmonella* Typhimurium CMCC 50115 strain | Lab stock |
| Δ*flgE* | a *flgE* deleted strain | This study |
| Δ*flgE/p**flgE* | (Δ*flgE*) complemented strain carrying pBad/gIIIA–*flgE* plasmid | This study |
| Δ*fliC* | a *fliC* deleted strain | This study |
| Δ*fliC*/p*fliC* | (Δ*fliC*) complemented strain carrying pBad/gIIIA–*fliC* plasmid | This study |
| ***Salmonella* strains** |  |  |
| *S.* Paratyphi A | a *Salmonella* Paratyphi A CMCC 50001 strain | Lab stock |
| *S*. Enteritidis | a *Salmonella* Enteritidis ATCC 13076 strain | Lab stock |
| *S.* Choleraesuis | a *Salmonella* Choleraesuis ATCC 10708 strain | Lab stock |
| *S*. Arizona | a *Salmonella* Arizona ATCC 13314 strain | Lab stock |
| **Plasmids** |  |  |
| pKD3 | FRT-cat-FRT, oriR6K; Ampr Cm^r.^ | Takara |
| pKD46 | λRed recombinase expression, Amp^r^ | Takara |
| pCP20 | Flp recombinase expression, Amp^r^ | Takara |
| pBad/gIIIA | Expression plasmid, containing arabinose-inducible promoter P_BAD_, Amp^r^ | Invitrogen |
| pBad/gIIIA-*flgE* | Δ*flgE* carrying pBad/gIIIA-*flgE* (Amp^r^) | This study |
| pBad/gIIIA-*fliC* | Δ*fliC* carrying pBad/gIIIA-*fliC* (Amp^r^) | This study |

**Table S 2 Strains and plasmids used in this study**

| **Primer name** | **Sequences (5'-3')** | **Description** |
| --- | --- | --- |
| *flgE*–F1 | TGGATCTGGGTACCTACGGCACCACCACACTCGACGAAGTTCGGCAAATAATCTAAGCCGTGTAGGCTGGAGCTGCTTC | Construction of Isogenic *flgE* mutant |
| *flgE*–R1 | TGGCTGGCCGCCCCCATGGCGGTATAAATTGCGTGATCCATTAAGCTATCCCGTCAGGCCATATGAATATCCTCCTTAG |  |
| *flgE*–F2 | CAGTTGGCGCAAATCAGTACC | Confirmed isogenic *flgE* mutant |
| *flgE*–R2 | GGCGGAATAAACCGTCATCG |  |
| *flgE*–F3 | TCCGAATTCATGTCTTTTTCTCAAGCGGTTAG | Construction of pBad/gIIIA–*flgE* |
| *flgE*–R3 | GCACTCGAGTTAGCGCAGGTTAACCAGCGTATT |  |
| *fliC*–D280-F1 | AGTGGCTGTTGTTCTTTTTC | Construction of Isogenic *fliC* mutant |
| *fliC*–D280-R1 | GAAGCAGCTCCAGCCTACACTCCGGCGATTGATTCACC |  |
| *fliC*–Cm-F2 | GGTGAATCAATCGCCGGAGTGTAGGCTGGAGCTGCTTC |  |
| *fliC*–Cm-R2 | AACATCAAGTTGTAATTGATCATATGAATATCCTCCTTAG |  |
| *fliC*–U280-F3 | CTAAGGAGGATATTCATATGATCAATTACAACTTGATGTT |  |
| *fliC*–U280-R3 | GTTTGATCCCACACCTAATG |  |
| *fliC*–D280-F1 | AGTGGCTGTTGTTCTTTTTC | Confirmed isogenic *fliC* mutant |
| *fliC*–U280-R3 | GTTTGATCCCACACCTAATG |  |
| *fliC*–F4 | TCCGAATTCATGGCACAAGTCATTAATACAAACA | Construction of pBad/gIIIA–*fliC* |
| *fliC*–R4 | GCACTCGAGTTAACGCAGTAAAGAGAGGACGTT |  |
| *fljB-*F | AATTGAACTCGACGAGCAAG | Confirmed *fljB* gene sequence |
| *fljB-*R | ATTCTGATATTAACGCCACC |  |


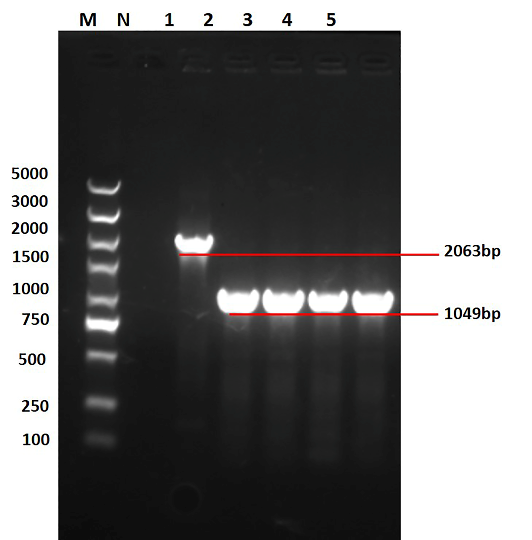


**Figure S 1 PCR results of gene knock-out strains**

M: Marker; N: Negative control; 1: *flgE* wild-type control; 2-5: PCR results of gene knock-out strains Δ*flgE*.

**
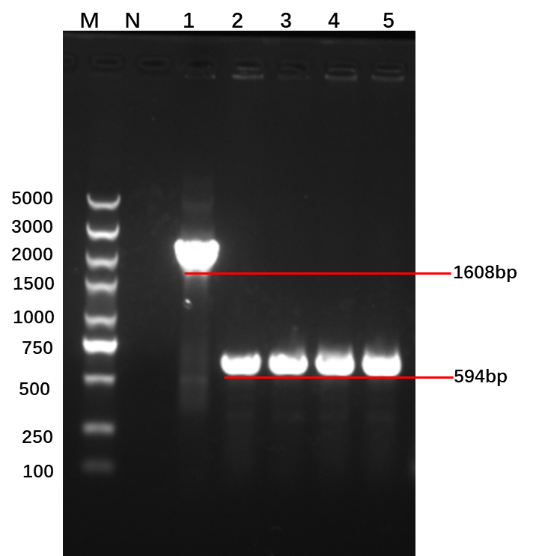
**

**Figure S 2** **PCR results of gene knock-out strains**

M: Marker; N: Negative control; 1: *fliC* wild-type control; 2-5: PCR results of gene knock-out strains Δ*fliC*.


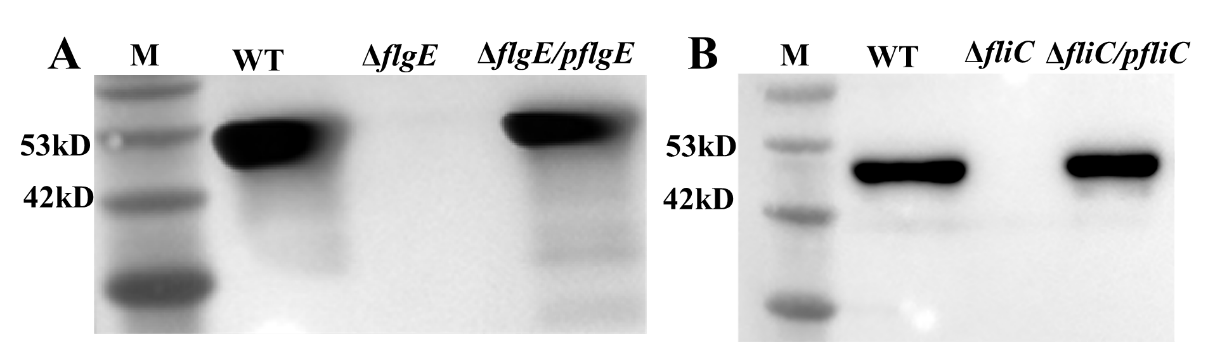


**Figure S 3 Flagellin deficiency was confirmed in the flagella mutants by immunoblot analysis**

M, Realband 3-color broad range protein marker (Sangon, China ), WT, wild-type.


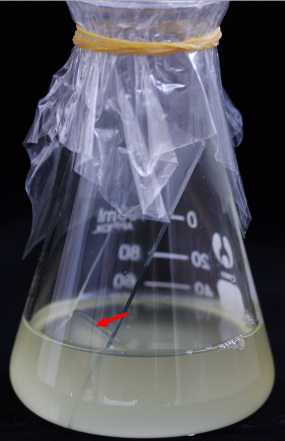


**Figure S 4 Biofilms formed on a sterile glass slide**

The arrow indicates the biofilms on air-liquid junction of a sterile glass slide.


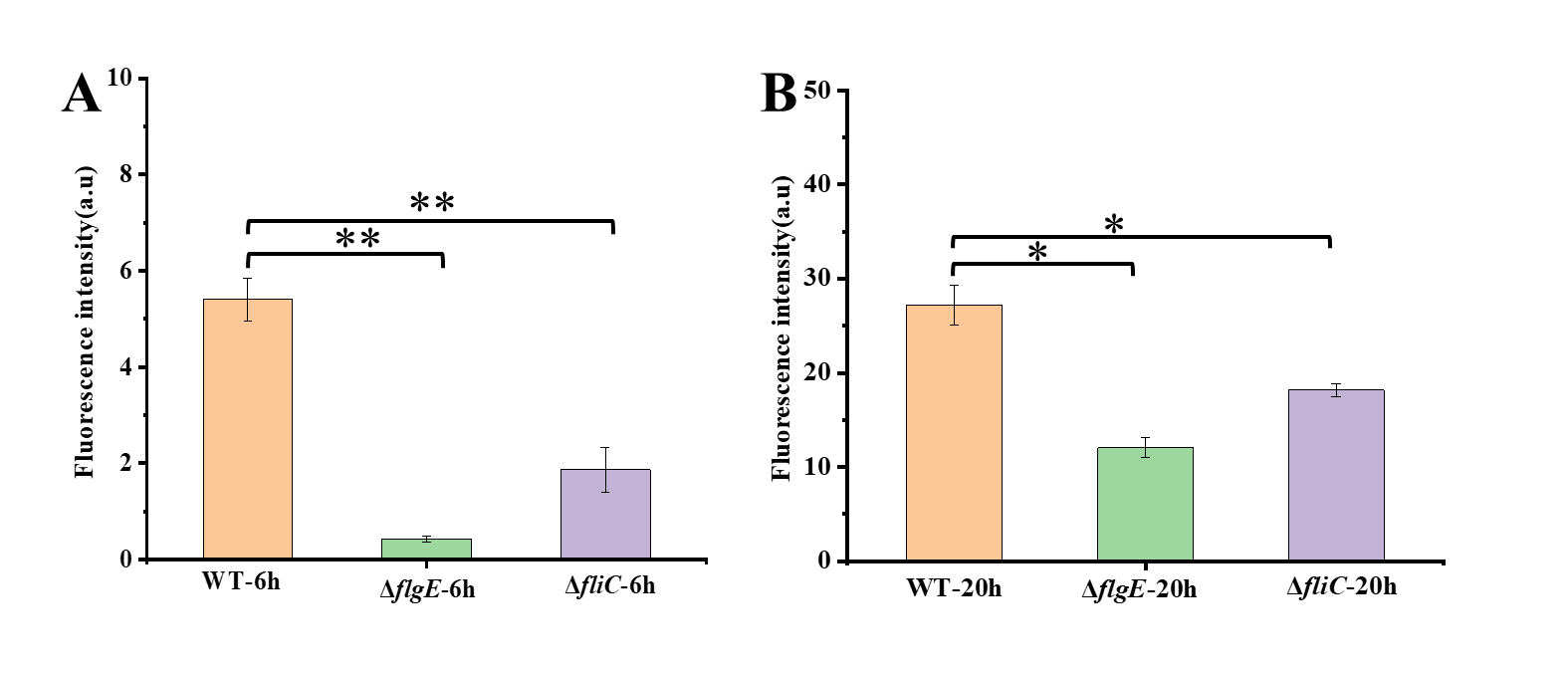


**Figure S 5** Statistical analysis of the fluorescence intensity of bacteria on a sterile glass slide for 6 h and 20 h by using Leica Application Suite X software. Error bars represent the standard deviation. The data were analyzed using the Student’s two-tailed t-test. * p < 0.05, ** p <0.005.


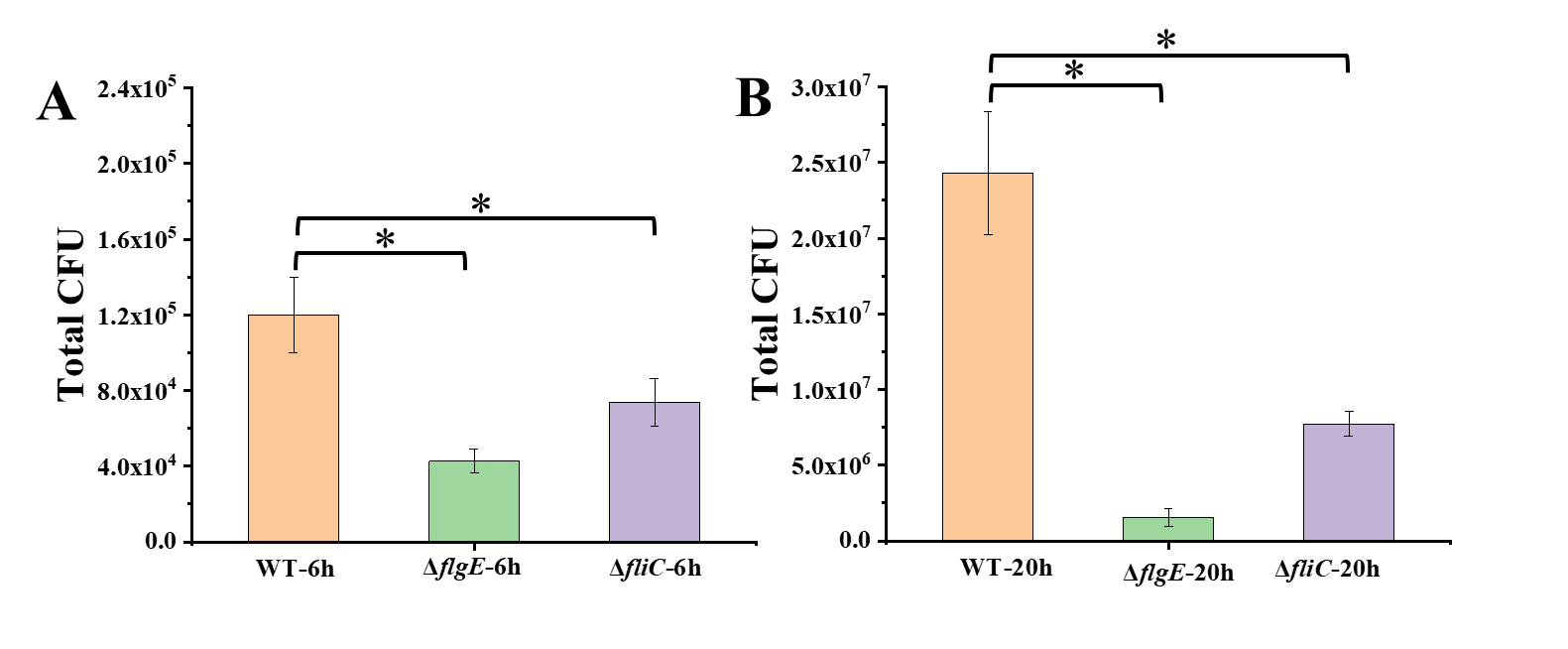


**Figure S 6** The number of colony forming units (CFU) on a sterile glass slide for 6 h and 20 h, which was calculated by using conversion factors determined from serial dilution plating after homogenization. Error bars represent the standard deviation. The data were analyzed using the Student’s two-tailed t-test. * p < 0.05 ** p <0.005.

**
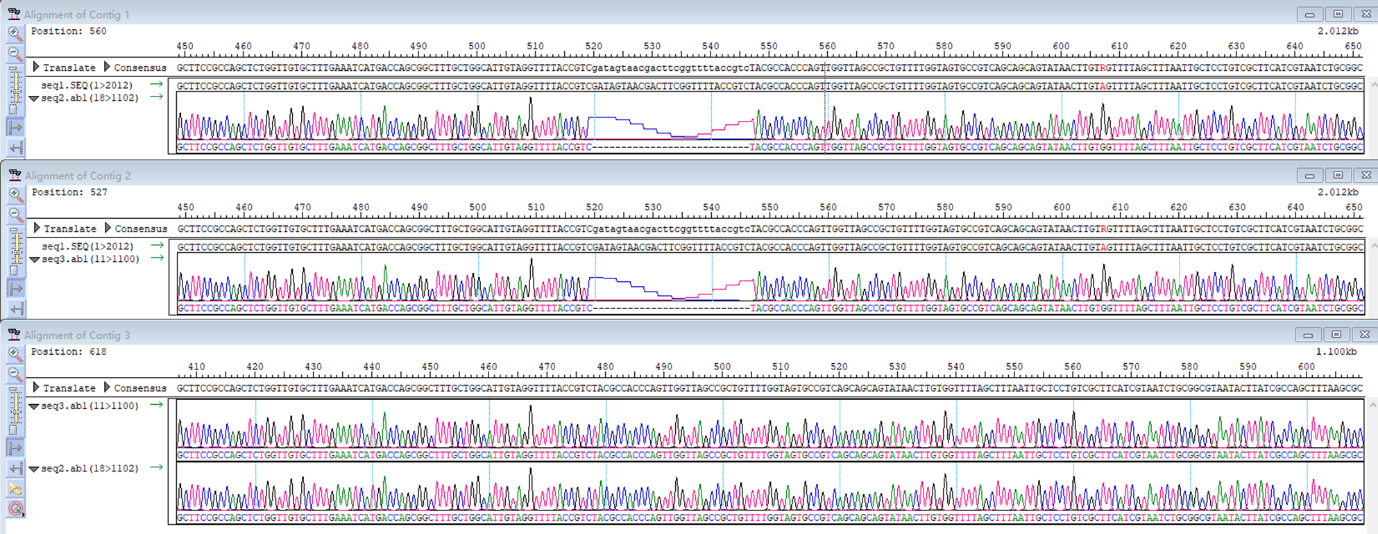
**

**Figure S 7 Analysis of the sequencing result**

Seq 1 was the *fljB* sequence of *S.* Typhimurium LT2 strain; Seq 2 was the *fljB* sequence of *S. Typhimurium CMCC 50115* strain；Seq 3 is the *fljB* sequence of the Δ*fliC* strain.
